# Supplementary material for: Use of artificial intelligence in obstetric and gynaecological diagnostics: a protocol for a systematic review and meta-analysis
Source: BMJ Open. 2024 May 8;14(5):e082287. doi: 10.1136/bmjopen-2023-082287 (PMC11086378; doi:10.1136/bmjopen-2023-082287)
Supplement: Supplementary data [file bmjopen-2023-082287supp001.pdf]

The use of artificial intelligence in obstetric and gynaecological diagnostics: a protocol for a systematic review and meta-analysis

The full search strategy to this systematic review are as follows:

Date range used (5 years, 10 years): May 2015 to date
Limits used (gender, article/study type, etc.): Female, human
Search terms and notes (full search strategy for database searches below):

Keyword terms

- Artificial Intelligence
  - machine learning or supervised learning or weak supervision or semi-supervised learning or active learning or structured prediction
  - structured learning or output learning or "learning to rank" or machine-learned ranking or unsupervised learning or reinforcement learning
  - neural network or artificial neural network or neural net
  - artificial intelligence or AI or deep learning
- Obstetrics and Gynaecology
  - "obstetrics and gynaecology" or "obstetrics and gynecology" or obstet* or gyn?ecol*
  - maternal-f?etal medicine or perinatology
  - "Reproductive endocrinology and infertility" or reproductive surgery
  - Gyn?ecological oncology or urogyn?ecology
  - Gyn?ecological oncology or urogyn?ecology or "female pelvic medicine and reconstructive surgery"
  - family planning or abortion or induced miscarriage or "termination of pregnancy" or (pregnanc* adj3 terminat*) or birth control or contracep*
- Diagnostic Imaging
  - medical imaging or diagnostic imaging
  - radiography or x-ray or fluoroscop* or projectional radiography or conventional radiography or plain radiography or roentgenography
  - magnetic resonance imaging or MRI
  - nuclear medicine or nucleology
  - medical ultrasound or medical ultrasonography or sonography or echography or ultrasound
  - computed tomography or CT Scan

Subject headings

	**MEDLINE**	**Embase**	**Cochrane**
Artificial Intelligence	Artificial Intelligence	Machine Learning	Artificial Intelligence
	Machine Learning	Semi Supervised Machine Learning	Machine Learning
	Deep Learning	Supervised Machine Learning	Deep Learning
	Supervised Machine Learning	Support Vector Machine	Supervised Machine Learning

	Support Vector Machine	Unsupervised Machine Learning	Support Vector Machine
	Unsupervised Machine Learning	Deep Learning	Unsupervised Machine Learning
	Sentiment Analysis	Artificial Intelligence	Sentiment Analysis
Obstetrics and Gynaecology	Gynecology	Gynecology	Gynecology
	Obstetrics	Obstetrics	Obstetrics
	Women's Health	Women's Health	Women's Health
	Maternal Health	Maternal Care	Maternal Health
	Reproductive Medicine	Reproduction	Reproductive Medicine
	Gynecology	Childbirth	Gynecology
	Reproductive Health	Pregnancy	Reproductive Health
	Sexual Health	Prenatal Development	Sexual Health
		Pseudopregnancy	
		Puerperium	
		Reproductive Health	
		Sexual Health	
Diagnostic Imaging	Diagnostic Imaging (exp)	Diagnostic Imaging (exp)	Diagnostic Imaging (exp)
		Computer Assisted Tomography	
		Radiography	
		Nuclear Magnetic Resonance Imaging (exp)	
		Nuclear Medicine	
		Echography	

The search strategy was devised and subsequently run in three resources across two platforms:

- Ovid
    - MEDLINE
    - Embase
- The Cochrane Library
-

Search History

Ovid MEDLINE(R) ALL <May 2015 to March 04, 2024>

1 (machine learning or supervised learning or weak supervision or semi-supervised learning or active learning or structured prediction).mp. [mp=title, book title, abstract, original title, name of substance word, subject heading word, floating sub-heading word, keyword heading word, organism supplementary concept word, protocol supplementary concept word, rare disease supplementary concept word, unique identifier, synonyms, population supplementary concept word, anatomy supplementary concept word]

2 (structured learning or output learning or "learning to rank" or machine-learned ranking or unsupervised learning or reinforcement learning).mp. [mp=title, book title, abstract, original title, name of substance word, subject heading word, floating sub-heading word, keyword heading word, organism supplementary concept word, protocol supplementary concept word, rare disease supplementary concept word, unique identifier, synonyms, population supplementary concept word, anatomy supplementary concept word]

3 (neural network or artificial neural network or neural net).mp. [mp=title, book title, abstract, original title, name of substance word, subject heading word, floating sub-heading word, keyword heading word, organism supplementary concept word, protocol supplementary concept word, rare disease supplementary concept word, unique identifier, synonyms, population supplementary concept word, anatomy supplementary concept word]

4 (artificial intelligence or AI or deep learning).mp. [mp=title, book title, abstract, original title, name of substance word, subject heading word, floating sub-heading word, keyword heading word, organism supplementary concept word, protocol supplementary concept word, rare disease supplementary concept word, unique identifier, synonyms, population supplementary concept word, anatomy supplementary concept word]

5 artificial intelligence/ or machine learning/ or deep learning/ or supervised machine learning/ or support vector machine/ or unsupervised machine learning/ or sentiment analysis/

6 1 or 2 or 3 or 4 or 5

7 ("obstetrics and gynaecology" or "obstetrics and gynecology" or obstet* or gyn?ecol*).mp. [mp=title, book title, abstract, original title, name of substance word, subject heading word, floating sub-heading word, keyword heading word, organism supplementary concept word, protocol supplementary concept word, rare disease supplementary concept word, unique identifier, synonyms, population supplementary concept word, anatomy supplementary concept word]

8 (maternal-f?etal medicine or perinatology).mp. [mp=title, book title, abstract, original title, name of substance word, subject heading word, floating sub-heading word, keyword heading word, organism supplementary concept word, protocol supplementary concept word, rare disease supplementary concept word, unique identifier, synonyms, population supplementary concept word, anatomy supplementary concept word]

9 ("Reproductive endocrinology and infertility" or reproductive surgery).mp. [mp=title, book title, abstract, original title, name of substance word, subject heading word, floating sub-heading word, keyword heading word, organism supplementary concept word, protocol supplementary concept word, rare disease supplementary concept word, unique identifier, synonyms, population supplementary concept word, anatomy supplementary concept word]

10 (Gyn?ecological oncology or urogyn?ecology).mp. [mp=title, book title, abstract, original title, name of substance word, subject heading word, floating sub-heading word, keyword heading word, organism supplementary concept word, protocol supplementary concept word, rare disease supplementary concept word, unique identifier, synonyms, population supplementary concept word, anatomy supplementary concept word]

11 (Gyn?ecological oncology or urogyn?ecology or "female pelvic medicine and reconstructive surgery").mp. [mp=title, book title, abstract, original title, name of substance word, subject heading word, floating sub-heading word, keyword heading word, organism supplementary concept word, protocol supplementary concept word, rare disease supplementary concept word, unique identifier, synonyms, population supplementary concept word, anatomy supplementary concept word]

12 (family planning or abortion or induced miscarriage or "termination of pregnancy" or (pregnanc* adj3 terminat*) or birth control or contracep*).mp. [mp=title, book title, abstract, original title, name of substance word, subject heading word, floating sub-heading word, keyword heading word, organism supplementary concept word, protocol supplementary concept word, rare disease supplementary concept word, unique identifier, synonyms, population supplementary concept word, anatomy supplementary concept word]

13 gynecology/ or obstetrics/

14 women's health/ or maternal health/

15 reproductive medicine/ or gynecology/

16 reproductive health/ or sexual health/

17 7 or 8 or 9 or 10 or 11 or 12 or 13 or 14 or 15 or 16

18 (medical imaging or diagnostic imaging).mp. [mp=title, book title, abstract, original title, name of substance word, subject heading word, floating sub-heading word, keyword heading word, organism supplementary concept word, protocol supplementary concept word, rare disease supplementary concept word, unique identifier, synonyms, population supplementary concept word, anatomy supplementary concept word]

19 (radiography or x-ray or flouroscop* or projectional radiography or conventional radiography or plain radiography or roentgenography).mp. [mp=title, book title, abstract, original title, name of substance word, subject heading word, floating sub-heading word, keyword heading word, organism supplementary concept word, protocol supplementary concept word, rare disease supplementary concept word, unique identifier, synonyms, population supplementary concept word, anatomy supplementary concept word]

20 (magnetic resonance imaging or MRI).mp. [mp=title, book title, abstract, original title, name of substance word, subject heading word, floating sub-heading word, keyword heading word, organism supplementary concept word, protocol supplementary concept word, rare disease supplementary concept word, unique identifier, synonyms, population supplementary concept word, anatomy supplementary concept word]

21 (nuclear medicine or nucleology).mp. [mp=title, book title, abstract, original title, name of substance word, subject heading word, floating sub-heading word, keyword heading word, organism supplementary concept word, protocol supplementary concept word, rare disease supplementary concept word, unique identifier, synonyms, population supplementary concept word, anatomy supplementary concept word]

22 (medical ultrasound or medical ultrasonography or sonography or echography or ultrasound).mp. [mp=title, book title, abstract, original title, name of substance word, subject heading word, floating sub-heading word, keyword heading word, organism supplementary concept word, protocol supplementary concept word, rare disease supplementary concept word, unique identifier, synonyms, population supplementary concept word, anatomy supplementary concept word]

23 (computed tomography or CT Scan).mp. [mp=title, book title, abstract, original title, name of substance word, subject heading word, floating sub-heading word, keyword heading word, organism supplementary concept word, protocol supplementary concept word, rare disease supplementary concept word, unique identifier, synonyms, population supplementary concept word, anatomy supplementary concept word]

24 exp Diagnostic Imaging/

25 18 or 19 or 20 or 21 or 22 or 23 or 24

26 6 and 17 and 25

27 (Animals/ not (Animals/ and Humans/)) or (exp animals/ not humans.sh.)

28 26 not 27

29 limit 28 to yr="2015 -Current"

Embase <May 2015 to 2024 March 04>

1 (machine learning or supervised learning or weak supervision or semi-supervised learning or active learning or structured prediction).mp. [mp=title, abstract, heading word, drug trade name, original title, device manufacturer, drug manufacturer, device trade name, keyword heading word, floating subheading word, candidate term word]

2 (structured learning or output learning or "learning to rank" or machine-learned ranking or unsupervised learning or reinforcement learning).mp. [mp=title, abstract, heading word, drug trade name, original title, device manufacturer, drug manufacturer, device trade name, keyword heading word, floating subheading word, candidate term word]

3 (neural network or artificial neural network or neural net).mp. [mp=title, abstract, heading word, drug trade name, original title, device manufacturer, drug manufacturer, device trade name, keyword heading word, floating subheading word, candidate term word]

4 (artificial intelligence or AI or deep learning).mp. [mp=title, abstract, heading word, drug trade name, original title, device manufacturer, drug manufacturer, device trade name, keyword heading word, floating subheading word, candidate term word]

5 machine learning/ or semi supervised machine learning/ or supervised machine learning/ or support vector machine/ or unsupervised machine learning/

6 deep learning/

7 artificial intelligence/

8 1 or 2 or 3 or 4 or 5 or 6 or 7

9 ("obstetrics and gynaecology" or "obstetrics and gynecology" or obstet* or gyn?ecol*).mp. [mp=title, abstract, heading word, drug trade name, original title, device manufacturer, drug manufacturer, device trade name, keyword heading word, floating subheading word, candidate term word]

10 (maternal-f?etal medicine or perinatology).mp. [mp=title, abstract, heading word, drug trade name, original title, device manufacturer, drug manufacturer, device trade name, keyword heading word, floating subheading word, candidate term word]

11 ("Reproductive endocrinology and infertility" or reproductive surgery).mp. [mp=title, abstract, heading word, drug trade name, original title, device manufacturer, drug manufacturer, device trade name, keyword heading word, floating subheading word, candidate term word]

12 (Gyn?ecological oncology or urogyn?ecology).mp. [mp=title, abstract, heading word, drug trade name, original title, device manufacturer, drug manufacturer, device trade name, keyword heading word, floating subheading word, candidate term word]

13 (Gyn?ecological oncology or urogyn?ecology or "female pelvic medicine and reconstructive surgery").mp. [mp=title, abstract, heading word, drug trade name, original title, device manufacturer, drug manufacturer, device trade name, keyword heading word, floating subheading word, candidate term word]

14 (family planning or abortion or induced miscarriage or "termination of pregnancy" or (pregnanc* adj3 terminat*) or birth control or contracep*).mp. [mp=title, abstract, heading word, drug trade name, original title, device manufacturer, drug manufacturer, device trade name, keyword heading word, floating subheading word, candidate term word]

15 gynecology/ or obstetrics/

16 women's health/

17 exp maternal care/

18 reproduction/ or childbirth/ or pregnancy/ or prenatal development/ or pseudopregnancy/ or puerperium/

19 reproductive health/

20 sexual health/

21 9 or 10 or 11 or 12 or 13 or 14 or 15 or 16 or 17 or 18 or 19 or 20

22 (medical imaging or diagnostic imaging).mp. [mp=title, abstract, heading word, drug trade name, original title, device manufacturer, drug manufacturer, device trade name, keyword heading word, floating subheading word, candidate term word]

23 (radiography or x-ray or flouroscop* or projectional radiography or conventional radiography or plain radiography or roentgenography).mp. [mp=title, abstract, heading word, drug trade name, original title, device manufacturer, drug manufacturer, device trade name, keyword heading word, floating subheading word, candidate term word]

24 (magnetic resonance imaging or MRI).mp. [mp=title, abstract, heading word, drug trade name, original title, device manufacturer, drug manufacturer, device trade name, keyword heading word, floating subheading word, candidate term word]

25 (nuclear medicine or nucleology).mp. [mp=title, abstract, heading word, drug trade name, original title, device manufacturer, drug manufacturer, device trade name, keyword heading word, floating subheading word, candidate term word]

26 (medical ultrasound or medical ultrasonography or sonography or echography or ultrasound).mp. [mp=title, abstract, heading word, drug trade name, original title, device manufacturer, drug manufacturer, device trade name, keyword heading word, floating subheading word, candidate term word]

27 (computed tomography or CT Scan).mp. [mp=title, abstract, heading word, drug trade name, original title, device manufacturer, drug manufacturer, device trade name, keyword heading word, floating subheading word, candidate term word]

28 exp diagnostic imaging/

29 exp computer assisted tomography/

30 radiography/

31 exp nuclear magnetic resonance imaging/

32 nuclear medicine/

33 exp echography/

34 22 or 23 or 24 or 25 or 26 or 27 or 28 or 29 or 30 or 31 or 32 or 33

35 8 and 21 and 34

36 (exp animal/ or nonhuman/) not exp human/

37 35 not 36

38 limit 37 to yr="2015 -Current"

Cochrane

Search

#1 machine learning or supervised learning or weak supervision or semi-supervised learning or active learning or structured prediction

#2 structured learning or output learning or "learning to rank" or machine-learned ranking or unsupervised learning or reinforcement learning

#3 neural network or artificial neural network or neural net

#4 artificial intelligence or AI or deep learning

#5 MeSH descriptor: [Artificial Intelligence] this term only

#6 MeSH descriptor: [Machine Learning] this term only

#7 MeSH descriptor: [Deep Learning] this term only

#8 MeSH descriptor: [Supervised Machine Learning] this term only

#9 MeSH descriptor: [Support Vector Machine] this term only

#10 MeSH descriptor: [Unsupervised Machine Learning] this term only

#11 MeSH descriptor: [Sentiment Analysis] explode all trees

#12 #1 OR #2 OR #3 OR #4 OR #5 OR #6 OR #7 OR #8 OR #9 OR #10 OR #11

#13 "obstetrics and gynaecology" or "obstetrics and gynecology" or obstet* or gyn?ecol*

#14 maternal-f?etal medicine or perinatology

#15 "Reproductive endocrinology and infertility" or reproductive surgery

#16 Gyn?ecological oncology or urogyn?ecology

#17 Gyn?ecological oncology or urogyn?ecology or "female pelvic medicine and reconstructive surgery"

#18 family planning or abortion or induced miscarriage or "termination of pregnancy" or (pregnanc* NEAR/3 terminat*)

#19 MeSH descriptor: [Gynecology] this term only

#20 MeSH descriptor: [Obstetrics] this term only

#21 MeSH descriptor: [Women's Health] this term only

#22 MeSH descriptor: [Maternal Health] this term only

#23 MeSH descriptor: [Reproductive Medicine] this term only

#24 MeSH descriptor: [Reproductive Health] this term only

#25 MeSH descriptor: [Sexual Health] this term only

#26 #13 OR #14 OR #15 OR #16 OR #17 OR #18 OR #19 OR #20 OR #21 OR #22 OR #23 OR #24 OR #25

#27 medical imaging or diagnostic imaging

#28 radiography or x-ray or flouroscop* or projectional radiography or conventional radiography or plain radiography or roentgenography

#29 magnetic resonance imaging or MRI

#30 nuclear medicine or nucleology

#31 medical ultrasound or medical ultrasonography or sonography or echography or ultrasound

#32 computed tomography or CT Scan

#33 MeSH descriptor: [Diagnostic Imaging] explode all trees

#34 #27 OR #28 OR #29 OR #30 OR #31 OR #32 OR #33

#35 #12 AND #26 AND #34

#36 Limit to custom date range 2015-2024 149 Cochrane Reviews / 16 Cochrane Protocols / 76 Cochrane Central Register Trials
