# Supplementary material for: Use of artificial intelligence in obstetric and gynaecological diagnostics: a protocol for a systematic review and meta-analysis
Source: BMJ Open. 2024 May 8;14(5):e082287. doi: 10.1136/bmjopen-2023-082287 (PMC11086378; doi:10.1136/bmjopen-2023-082287)
Supplement: Author's manuscript [file bmjopen-2023-082287.draft_revisions.pdf]

BMJ Open is committed to open peer review. As part of this commitment we make the peer review history of every article we publish publicly available.

When an article is published we post the peer reviewers' comments and the authors' responses online. We also post the versions of the paper that were used during peer review. These are the versions that the peer review comments apply to.

The versions of the paper that follow are the versions that were submitted during the peer review process. They are not the versions of record or the final published versions. They should not be cited or distributed as the published version of this manuscript.

BMJ Open is an open access journal and the full, final, typeset and author-corrected version of record of the manuscript is available on our site with no access controls, subscription charges or pay-per-view fees (http://bmjopen.bmj.com).

If you have any questions on BMJ Open's open peer review process please email info.bmjopen@bmj.com

The use of artificial intelligence in obstetric and gynaecological diagnostics: a protocol for a systematic review and meta-analysis

Journal:	*BMJ Open*
Manuscript ID	bmjopen-2023-082287
Article Type:	Protocol
Date Submitted by the Author:	19-Nov-2023

Complete List of Authors:	Chaurasia, Anjalee; Imperial College London, School of Medicine<br>Curry, Georgia; Imperial College London, School of Medicine<br>Zhao, Yi; Imperial College London, School of Medicine<br>Dawoodbhoy, Fatema; Barking Havering and Redbridge Hospitals NHS Trust<br>Green, Jennifer; North West Anglia NHS Foundation Trust, Department of Obstetrics & Gynaecology<br>Vaninetti, Matilde; Cambridge University Hospitals NHS Foundation Trust<br>Shah, Nishel; Chelsea and Westminster Hospital, Department of Metabolism, Digestion and Reproduction; Chelsea and Westminster Hospital NHS Foundation Trust<br>Greer, Orene; Chelsea and Westminster Hospital, Department of Metabolism, Digestion and Reproduction; Chelsea and Westminster Hospital NHS Foundation Trust

Keywords:	OBSTETRICS, GYNAECOLOGY, Systematic Review, Meta-Analysis

SCHOLARONE™
Manuscripts

The use of artificial intelligence in obstetric and gynaecological diagnostics: a protocol for a systematic review and meta-analysis

Georgia Curry[1], Anjalee Chaurasia[1], Yi Zhao[1], Fatema Dawoodbhoy[2], Jennifer Green[3] Matilde Vaninetti[4], Nishel Shah[5,6], Orene Greer[5,6]

Georgia Curry and Anjalee Chaurasia are the joint first authors*

[1] Imperial College London School of Medicine, London, United Kingdom

[2] Barking, Havering and Redbridge NHS Trust, United Kingdom

[3] Department of Obstetrics & Gynaecology, Hinchingbrooke Hospital, Northwest Anglia NHS Foundation Trust, United Kingdom.

[4] Cambridge University Hospitals NHS Foundation Trust, United Kingdom

[5] Imperial College London, Department of Metabolism, Digestion and Reproduction, Chelsea and Westminster Hospital, London, United Kingdom.

[6] Chelsea & Westminster NHS Foundation Trust, 369 Fulham Road, London, United Kingdom

Correspondence to: Georgia Curry; Anjalee Chaurasia

Imperial College London, Faculty of Medicine

Ayrton Rd, South Kensington, London SW7 5NH

glc17@ic.ac.uk ; aac3018@ic.ac.uk

Ethics: No ethical considerations apply.

For peer review only

Word count: 1874

ABSTRACT

Introduction: Emerging developments in applications of artificial intelligence (AI) in healthcare offer the opportunity to improve diagnostic capabilities in obstetrics and gynaecology (O&G), ensuring early detection of pathology, optimal management and improving survival. Consensus on a robust AI healthcare framework is crucial for standardizing protocols that promote data privacy and transparency, minimize bias, and ensure patient safety. Here, we describe the study protocol for a systematic review and meta-analysis to evaluate current applications of AI in O&G diagnostics with consideration of reporting standards used and their ethical implications. Overall, this review will aid clinicians' understanding of AI in healthcare and its potential ethical downfalls, along with what is required in a rigorous AI model. This protocol is written following the Preferred Reporting Items for Systematic Review and Meta-Analysis Protocols (PRISMA-P) 2015 checklist.

Methods and analysis: The study objective is to explore the current application of AI in O&G diagnostics and assess the reporting standards used in these studies. Electronic bibliographic databases MEDLINE, PubMed, EMBASE and Cochrane will be searched. Study selection, data extraction, and subsequent narrative synthesis and meta-analyses will be carried out following the PRISMA-P guidelines. Included papers will be English-language full-text articles from May 2015 to August 2023 which provide original data. Papers must use AI as the predictive method, focusing on improving O&G diagnostic outcomes.

We will evaluate the reporting standards including the risk of bias, lack of transparency and consider the ethical implications and potential harm to patients. Outcome measures will involve assessing the included studies against gold standard criteria for robustness of model development (Transparent reporting of a multivariable prediction model for individual prognosis or diagnosis (TRIPOD), model predictive performance, model risk of bias and applicability (Prediction model Risk Of Bias Assessment Tool (PROBAST) and study reporting (Consolidated Standards of Reporting Trials-Artificial Intelligence) CONSORT-AI guidance.

Ethics and dissemination: Ethical approval is not required for this systematic review. Findings will be shared through peer-reviewed publications. There will be no patient or public involvement in this study.

For peer review only

PROSPERO registration number: CRD42022357024

Strengths and limitations of the study:

- This will be the first comprehensive systematic review and meta-analysis of the ethical considerations of the application of AI in O&G diagnostics.
- Statistical analysis will be performed to determine the effect of heterogeneity in the literature.
- Heterogeneity of study methods may impact the strength of the conclusions.
- The analysis may be limited if a small study number meets the inclusion criteria.

For peer review only

BACKGROUND

Access to accurate diagnostics is fundamental to reducing morbidity and mortality. However, common diagnostic tools used in areas of obstetrics and gynaecology (O&G) have their limitations. Many of the diagnostics are performed using ultrasound, which is subjective and prone to error (1,2). For instance, inter and intra-operator variation in foetal birth weight estimates and discrepancies in nuchal translucency are acknowledged (4-6). Another example is in the tools used to predict preterm birth, a major cause of neonatal death worldwide.

Additional work is required to improve our ability to accurately predict it and subsequently improve clinical outcomes (7,8); particularly as much of the resultant mortality might be prevented with cost-effective interventions (9-12). Thus, emerging technological AI developments in medicine may offer an opportunity to improve diagnostic capabilities in O&G (3).

Artificial intelligence (AI) uses multifactorial data to build connections and learn. Such data can take the form of images or numeric values obtained from existing databases or laboratory tests (13). For example, machine learning models can be applied to a series of medical images to extract key features associated with specific pathologies and this can be used to increase the accuracy of detecting gynaecological malignancy e.g., malignant epithelial ovarian tumours (15). It has been suggested that AI has the potential to perform problem-solving at a greater efficiency than humans (14).

However, the development in AI technology requires parallel development of rigorous protocols, which can be applied across AI applications, to ensure data privacy, transparency, safety and fairness, as well as to minimise bias. The importance of improved governance of AI research can be demonstrated by the example of an algorithm which was developed to determine patient health risks for approximately 200 million individuals in the US each year (16). It was shown to exhibit racial bias, with the choice of label used to train this data resulting in fewer black patients receiving the care they needed (16).

The implementation of this new technology, if unregulated, has the potential to cause personal and societal harm, with risk to patient safety (18). Foremost, AI algorithms may be subject to predictive error due to the training data set used. This may result in incorrect clinical findings and subsequent under or over treatment, unknown to both clinician and patient (19). Therefore, it is important that researchers developing AI models acknowledge the ethical implications AI may pose for healthcare and adhere to recommended standards for reporting of their methods and data. We aim to evaluate current practice and highlight where work is required to improve this. Thus, we will systematically review existing studies, assessing reporting standards, performance, model development and performance, and adherence to 'gold standard' criteria.

METHODS AND ANALYSIS

For peer review only

The protocol is registered with the international Prospective Register of Systematic Reviews (PROSPERO), registration number CRD42022357024. The protocol and the completed systematic review will be reported using the Preferred Reporting Items for Systematic Reviews and Meta-analyses for protocols (PRISMA-P) (20). Any post-registration changes will be included in the International prospective register of systematic reviews record (PROSPERO)(21).

Search Methodology

The search will be carried out using Medical Literature Analysis and Retrieval System Online (MEDLINE), PubMed, Excerpta Medica Database (EMBASE) and Cochrane. The search strategy will include Medical Subject Headings (MESH) terms, free text and Boolean operators. We will search using the terms "obstetrics" and "gynaecology", multiple synonyms of "artificial intelligence" including "machine learning", "deep learning" and "algorithm", along with "diagnostics", "prognostics" and their variations. The search strategy will be adapted to the specific database. A manual search of the grey literature will also be performed. The studies included will only be in the English language, published from May 2015 to March 2023(24).

Study selection and data extraction

The studies will be uploaded to a systematic review study management software, for example Rayyan (25), to remove duplicates. The studies will be screened by 2 independent reviewers, comparing the titles and abstracts to the inclusion criteria. The same process will then be carried out for the full text of the selected studies. Any disagreements between the reviewers will be resolved via discussion or following discussion with an additional reviewer. Study exclusion criteria will be recorded and reported in the review. The references of manuscripts identified from the search will also be screened for studies suitable for inclusion. A PRISMA flow chart of the search and study selection will be included in the report(30).

For peer review only

Inclusion criteria

This systematic review will include peer-reviewed studies that use patient data for predictive outcomes. To be included in the analysis, studies must use AI as the predictive method, focusing on improving O&G diagnostic outcomes and be written in English.

Exclusion criteria

Non-human studies and papers that do not use patient data for predictive outcomes. Review articles, commentaries, abstracts, meta-analyses, case reports, and expert opinions, will be excluded. Literature that does not discuss AI in O&G diagnostics and papers not published in the English language will also be excluded.

Data extraction

Data will be extracted from the studies using Excel. The data extracted will include the Year of publication, Study authors, Study design, Patient population, Study size, the O&G pathology evaluated, the diagnostic technique, type of AI model, reporting system for example TRIPOD-AI, the performance measurements used, the method of clinical deployment of the AI model and whether ethical approval was granted for each study. Table 1 summarises the data to be extracted.

Table 1 Data collection items

**Item no.**	Data title	Data type
**1**	Year of publication	Study characteristic
**2**	Study authors	Study characteristic
**3**	Study design	Study characteristic

For peer review only

**4**	Patient population	Demographics
**5**	Study size	Demographics
**6**	Target O&G pathology	Methodology
**7**	Diagnostic technique	Methodology
**8**	Type of AI model	Outcome
**9**	Reporting system used	Outcome
**10**	Method of illustrating diagnostic performance	Outcome
**11**	Method of model deployment	Outcome
**12**	Whether ethical approval was granted for each study (Y/N)	Outcome

O&G- Obstetrics and gynaecology. AI- artificial intelligence.

Endpoints

The primary endpoint is a demonstration of the application of current recommended standards for the development and reporting of AI primary research involving O&G diagnostics (relative to CONSORT-AI, Transparent reporting of a multivariable prediction model for individual prognosis or diagnosis (TRIPOD) (22) and Prediction model Risk Of Bias Assessment Tool (PROBAST) guidelines) (23). We will summarise these findings in a table.

Meta-analysis

If there are sufficient papers illustrating the diagnostic performance of the AI models for diseases relating to the field of obstetrics and gynaecology, a meta-analysis will be conducted. Model performance measures, including sensitivity, specificity and the area under the receiver operating curve (AUROC) values for specific O&G endpoints will be compared in the meta-analysis. The pooled sensitivity and specificity can be compared to the

For peer review only

reference standards and reported. If any of the required data has not been reported, we will contact the study authors to request data sharing. If studies present a different metric for model performance, these will also be included in the report.

A 2x2 contingency table containing true positive, false positive, true negative, false negative will be created for the sensitivity and specificity of each study and compared using bivariate analysis. These will subsequently be plotted on forest plots and summary receiver operating characteristic (sROC) curve plots. The sROC will be calculated with the individual AUC.

Interstudy variation and heterogeneity will be displayed using I^2. To obtain pooled estimates, we will use a random-effects model. Leave-one-out analyses will be used to detect outliers, and the model will be changed accordingly. To control for study variance, we will use an untransformed, logit and double-arcsine transformed proportions, and normality will be assessed using the Shapiro-Wilk test and density plots. Ratios resembling normal distributions will be used. Analysis and visualization will be carried out using R statistical environment (V.3.6.1, 2019-07-05).

Risk of bias in individual studies

PROBAST will be used to assess the included studies for bias and applicability(23). It is divided into 4 domains: participants, predictors, outcome, and analysis. It has 20 questions that help to judge bias. Bias occurs when the shortcomings in the study design, conduct or analysis impact predictive performance. It will give an idea of the reliability of the data produced and the appropriateness of the model for diagnosis and prognosis. If studies with high bias are included, the impact and reasons for this will be discussed.

Patient and Public involvement

There will be no patient or public involvement in this study.

Trial status

For peer review only

- Preliminary searches: started
- Piloting of the study selection process: started
- Formal screening: not started
- Data extraction: not started
- Risk of bias assessment: not started
- Data analysis: not started

For peer review only

Draft search strategy

(("Machine Learning "[MeSH] OR "Artificial Intelligence "[MeSH] OR "Deep Learning"[MeSH] OR "Neural Networks (Computer)"[MeSH] OR machine learning OR artificial intelligence OR deep learning OR neural network) AND ("Obstetrics"[MeSH] OR "Gynecology"[MeSH] OR obstetrics OR gynecology OR gynaecology) AND (ultrasound OR "Diagnostic Imaging"[MeSH] OR diagnostics)) AND "Humans"[MeSH] AND "2015/01/01"[PDAT] : "3000"[PDAT] AND English[lang]

ETHICS AND DISSEMINATION

There are no ethical concerns as the review does not require consent or subject participation. Dissemination of the protocol and systematic review will be done through a peer-reviewed journal.

Author Affiliations

[1] Imperial College London School of Medicine, London, UK

[2] Barking, Havering and Redbridge NHS Trust

[3] Department of Obstetrics & Gynaecology, Hinchingbrooke Hospital, Northwest Anglia NHS Foundation Trust, UK.

[4] Imperial College London, Department of Metabolism, Digestion and Reproduction, Chelsea and Westminster Hospital, London, United Kingdom.

[5] Cambridge University Hospitals NHS Foundation Trust

Email: Anjalee Chaurasia: aac3018@ic.ac.uk, Georgia Curry: glc17@ic.ac.uk, Yi Zhao: yi.zhao18@ic.ac.uk

Contributors: The authors' contribution includes, but is not limited to, the following: GC, AC and YZ drafted the manuscript and created the study concept. OG, NS, FD, JG and MV provided supervision and guidance during the study. All authors reviewed and approved the manuscript in its current form. GC and AC are the guarantors of this work.

Funding: All authors acknowledge the funding from the Imperial College London Open Access Fund

Competing Interests: None declared.

Patient consent for publication: Not required

Provenance and peer review: Not commissioned; externally peer-reviewed

Open access This is an open-access article distributed in accordance with the Creative Commons Attribution 4.0 Unreported (CC BY 4.0) license which permits others to copy, redistribute, remix, transform and build upon this work for any purpose, provided the original work is properly cited, a link to the license is given, and indication of whether changes were made. See https://creativecommons.org/licenses/by/4.0/.

For peer review only

ORCHID IDs

- Georgia Curry https://orcid.org/0000-0002-2888-3859
- Anjalee Chaurasia https://orchid.org/0000-0003-3924-9201
- Yi Zhao https://orcid.org/0000-0002-4563-4344
- Fatema Dawoodbhoy https://orchid.org/0000-0002-7255-688X
- Nishel Shah https://orcid.org/0000-0002-5694-9915
- Orene Greer https://orcid.org/0000-0002-4940-0828

For peer review only

REFERENCES

1. Sotiriadis A, Odibo AO. Systematic error and cognitive bias in obstetric ultrasound. Ultrasound in Obstetrics & Gynecology. 2019 April; 53(4):431–5. DOI: 10.1002/uog.20232.

2. Dudley NJ. The management of error in ultrasound fetal growth monitoring. Ultrasound. 2021 February; 29(1):4-9 DOI: 742271X2094574.

3. Kim YH, Cho GJ, Kwon HS. Applications of artificial intelligence in obstetrics. Ultrasonography. 2023 January; 42(1): 2-9. DOI: 10.14366/usg.22063.

4. Chen Z, Liu Z, Du M, Want Z. Artificial Intelligence in Obstetric Ultrasound: An Update and Future Applications. Frontiers in Medicine. 2021 August; 8(1). DOI: 10.3389/fmed.2021.733468.

5. Milner J, Arezina J. The accuracy of ultrasound estimation of fetal weight in comparison to birth weight: A systematic review. Ultrasound. 2018 February; 26(1): 32-41. DOI: 10.1177/1742271x17732807.

6. Benacerraf BR, Minton KK, Benson CB, Scarborough NA, Wax J, Abuhamad AZ. Proceedings: Beyond Ultrasound First Forum on improving the quality of ultrasound imaging in

obstetrics and gynecology. American Journal of Obstetrics & Gynecology. 2018 January; 218(1): 19-28. DOI: 10.1016/j.ajog.2017.06.033.

7. Stock SJ, Horne M, Bruijn M, White H, Boyd KA, Heggie R, et al. Development and validation of a risk prediction model of preterm birth for women with preterm labour symptoms (the QUIDS study): A prospective cohort study and individual participant data meta-analysis. PLoS Med. 2021 July; 18(7). DOI: 10.1371/journal.pmed.1003686.

8. Suff N, Story L, Shennan A. The prediction of preterm delivery: What is new? Semin Fetal Neonatal Med. 2019 February; 24(1): 27-32. DOI: 10.1016/j.siny.2018.09.006.

9. Lee KS, Ahn KH. Application of Artificial Intelligence in Early Diagnosis of Spontaneous Preterm Labor and Birth. Diagnostics (Basel). 2020 September; 10(9): 733. DOI: 10.3390/diagnostics10090733.

10. Iftikhar P, Kuijpers MV, Khayyat A, de Gouveia De Sa M. Artificial Intelligence: A New Paradigm in Obstetrics and Gynecology Research and Clinical Practice. Cureus. 2020 February; 12(2): e7124. DOI: 10.7759/cureus.7124.

11. Tucker K, Bhardwag NR, Clark E, Espey E. Delayed diagnosis and management of second trimester abdominal pregnancy. Case Reports. 2017 September; 2017(1). DOI: 10.1136/bcr-2017-221433.

12. Harrison MS, Goldenberg RL. Global burden of prematurity. Elsevier. 2016 Apr; 21(2): 74-79. DOI: 10.1016/j.siny.2015.12.007

13. O'Leary D. Artificial Intelligence and Big Data. IEEE Intelligent Systems. 2013 March; 28(2): 96-99. DOI: 10.1109/MIS.2013.39

14. Davenport T, Kalakota R. The potential for artificial intelligence in healthcare. Future Healthcare Journal. 2019 June; 6(2): 94-98. DOI: 10.7861/futurehosp.6-2-94.

15. Li Y, Jian J, Pickhardt PJ, Ma F, Xia W, Li H, et al. MRI- Based Machine Learning for Differentiating Borderline From Malignant Epithelial Ovarian Tumours: A Multicenter Study.

For peer review only

Journal of Magnetic Resonance Imaging. 2020 February; 52(3): 897-904. DOI: 10.1002/jmri.27084.

16. Obermeyer Z, Powers B, Vogell C, Mullainathan S. Dissecting racial bias in an algorithm used to manage the health of populations. Science (American Association for the Advancement of Science). 2019 October; 366(6464): 447-453. DOI: 10.1126/science.aax2342.

17. Haibe-Kains B, Adam GA, Hosny A, Khodakarami F, Waldron L. Transparency and reproducibility in artificial intelligence. Nature. 2020 October; 586: E14-E16. DOI: 10.1038/s41586-020-2766-y.

18. Kim DW, Jang HY, Kim KW, Shin Y, Park SH. Design Characteristics of Studies Reporting the Performance of Artificial Intelligence Algorithms for Diagnostic Analysis of Medical Images: Results from Recently Published Papers. Korean Journal of Radiology. 2019 Mar; 20(3): 405-410. DOI: 10.3348/kjr.2019.0025.

19. Kristiansen TB, Kristensen K, Uffelmann J, Brandslund I. Erroneous data: The Achilles' heel of AI and personalized medicine. Frontiers in Digital Health. 2022 July; 4. DOI: 10.3389/fdgth.2022.862095.

20. Moher D, Shamseer L, Clarke M, Ghersi D, Liberati A, Petticrew M. Preferred reporting items for systematic review and meta-analysis protocols (PRISMA-P) 2015 statement. Systematic Reviews. 2015 January; 4. DOI: 10.1186/2046-4053-4-1.

21. PROSPERO [Online]. York.ac.uk. 2019. Available from: https://www.crd.york.ac.uk/prospero/

22. Collins GS, Reitsma JB, Altman DG, Moons K. Transparent reporting of a multivariable prediction model for individual prognosis or diagnosis (TRIPOD): the TRIPOD Statement. BMC Medicine. 2015 January; 13(1): 1. DOI: 10.1186/s12916-014-0241-z.

23. Wolff RF, Moons KGM, Riley RD, Whiting PF, Westwood M, Collins GS, et al. PROBAST: A Tool to Assess the Risk of Bias and Applicability of Prediction Model Studies. Annals of Internal Medicine. 2019 Jan; 170(1): 51. DOI: 10.7326/m18-1376.

For peer review only

24. LeCun Y, Bengio Y, Hinton G. Deep Learning. Nature. 2015 May; 521(7553): 436-444. DOI: 10.1038/nature14539.

25. Rayyan. rayyan.ai. [Online]. [cited 2023 January 22]. Available from: https://www.rayyan.ai/.

26. Dhombres F, Bonnard J, Baily K, Maurice P, Papageorghiou AT. Contributions of Artificial Intelligence Reported in Obstetrics and Gynecology Journals: Systematic Review. Journal of Medical Internet Research. 2022 April; 24(4): e35465. DOI: 10.2196/35465.

27. Meszaros J, Minari J, Huys I. The future regulation of artificial intelligence systems in healthcare services and medical research in the European Union. Frontiers in Genetics. 2022 October; 13. DOI: 10.3389/fgene.2022.927721.

28. Naik N, Hameed B, Shetty DK, Swain D, Shah M, Paul R, et al. Legal and Ethical Consideration in Artificial Intelligence in Healthcare: Who Takes Responsibility? Frontiers in Surgery. 2022 March; 9: 862322. DOI: 10.3389/fsurg.2022.862322.

29. Leslie D. Understanding artificial intelligence ethics and safety. [Online].; 2019 [cited 2023 January 22]. Available from: https://www.turing.ac.uk/sites/default/files/2019-06/understanding_artificial_intelligence_ethics_and_safety.pdf

30. PRISMA. PRISMA [Online]. Prisma-statement.org. 2015. Available from: https://www.prisma-statement.org

For peer review only

The use of artificial intelligence in obstetric and gynaecological diagnostics: a protocol for a systematic review and meta-analysis

The search terms for this systematic review are as follows:

(("Machine Learning "[MeSH] OR "Artificial Intelligence "[MeSH] OR "Deep Learning"[MeSH] OR "Neural Networks (Computer)"[MeSH] OR machine learning OR artificial intelligence OR deep learning OR neural network) AND ("Obstetrics"[MeSH] OR "Gynecology"[MeSH] OR obstetrics OR gynecology OR gynaecology) AND (ultrasound OR "Diagnostic Imaging"[MeSH] OR diagnostics)) AND "Humans"[MeSH] AND "2015/01/01"[PDAT] : "3000"[PDAT] AND English[lang]

Searches will be conducted on the following databases:

PubMed, MEDLINE, Embase, and Cochrane databases

For peer review only

PRISMA-P 2015 Checklist

This checklist has been adapted for use with protocol submissions to *Systematic Reviews* from Table 3 in Moher D et al: Preferred reporting items for systematic review and meta-analysis protocols (PRISMA-P) 2015 statement. *Systematic Reviews* 2015 **4**:1

Section/topic	#	Checklist item	Information reported: Yes	Information reported: No	Line number(s)
**ADMINISTRATIVE INFORMATION**					
**Title**					
Identification	1a	Identify the report as a protocol of a systematic review	☒	☐	1
Update	1b	If the protocol is for an update of a previous systematic review, identify as such	☐	☒	
**Registration**	2	If registered, provide the name of the registry (e.g., PROSPERO) and registration number in the Abstract	☒	☐	53
**Authors**					
Contact	3a	Provide name, institutional affiliation, and e-mail address of all protocol authors; provide physical mailing address of corresponding author	☒	☐	4-20
Contributions	3b	Describe contributions of protocol authors and identify the guarantor of the review	☒	☐	220-223
**Amendments**	4	If the protocol represents an amendment of a previously completed or published protocol, identify as such and list changes; otherwise, state plan for documenting important protocol amendments	☒	☐	228-232
**Support**					
Sources	5a	Indicate sources of financial or other support for the review	☒	☐	224
Sponsor	5b	Provide name for the review funder and/or sponsor	☒	☐	224
Role of sponsor/funder	5c	Describe roles of funder(s), sponsor(s), and/or institution(s), if any, in developing the protocol	☒	☐	224
**INTRODUCTION**					
**Rationale**	6	Describe the rationale for the review in the context of what is already known	☒	☐	70-90
**Objectives**	7	Provide an explicit statement of the question(s) the review will address with reference to participants, interventions, comparators, and outcomes (PICO)	☒	☐	36-49
**METHODS**					

BioMed Central
The Open Access Publisher

For peer review only - http://bmjopen.bmj.com/site/about/guidelines.xhtml

Section/topic	#	Checklist item	Information reported Yes	No	Line number(s)
**Eligibility criteria**	8	Specify the study characteristics (e.g., PICO, study design, setting, time frame) and report characteristics (e.g., years considered, language, publication status) to be used as criteria for eligibility for the review	☒	☐	131-133
**Information sources**	9	Describe all intended information sources (e.g., electronic databases, contact with study authors, trial registers, or other grey literature sources) with planned dates of coverage	☒	☐	109-110
**Search strategy**	10	Present draft of search strategy to be used for at least one electronic database, including planned limits, such that it could be repeated	☒	☐	198-202
STUDY RECORDS					
Data management	11a	Describe the mechanism(s) that will be used to manage records and data throughout the review	☒	☐	153-173
Selection process	11b	State the process that will be used for selecting studies (e.g., two independent reviewers) through each phase of the review (i.e., screening, eligibility, and inclusion in meta-analysis)	☒	☐	121-128
Data collection process	11c	Describe planned method of extracting data from reports (e.g., piloting forms, done independently, in duplicate), any processes for obtaining and confirming data from investigators	☒	☐	121-128
**Data items**	12	List and define all variables for which data will be sought (e.g., PICO items, funding sources), any pre-planned data assumptions and simplifications	☒	☐	142-148
**Outcomes and prioritization**	13	List and define all outcomes for which data will be sought, including prioritization of main and additional outcomes, with rationale	☒	☐	142-148
**Risk of bias in individual studies**	14	Describe anticipated methods for assessing risk of bias of individual studies, including whether this will be done at the outcome or study level, or both; state how this information will be used in data synthesis	☒	☐	176-181
DATA					
**Synthesis**	15a	Describe criteria under which study data will be quantitatively synthesized	☒	☐	157-163
	15b	If data are appropriate for quantitative synthesis, describe planned summary measures, methods of handling data, and methods of combining data from studies, including any planned exploration of consistency (e.g., I^2, Kendall's tau)	☒	☐	164-173
	15c	Describe any proposed additional analyses (e.g., sensitivity or subgroup analyses, meta-regression)	☒	☐	164-173
	15d	If quantitative synthesis is not appropriate, describe the type of summary planned	☐	☐	
**Meta-bias(es)**	16	Specify any planned assessment of meta-bias(es) (e.g., publication bias across studies, selective reporting within studies)	☐	☒	
**Confidence in cumulative evidence**	17	Describe how the strength of the body of evidence will be assessed (e.g., GRADE)	☐	☒	

BioMed Central
The Open Access Publisher

For peer review only - http://bmjopen.bmj.com/site/about/guidelines.xhtml

BMJ Open

The use of artificial intelligence in obstetric and gynaecological diagnostics: a protocol for a systematic review and meta-analysis

Journal:	*BMJ Open*
Manuscript ID	bmjopen-2023-082287.R1
Article Type:	Protocol
Date Submitted by the Author:	26-Mar-2024
Complete List of Authors:	Chaurasia, Anjalee; Imperial College London, School of Medicine<br>Curry, Georgia; Imperial College London, School of Medicine<br>Zhao, Yi; Imperial College London, School of Medicine<br>Dawoodbhoy, Fatema; Barking Havering and Redbridge Hospitals NHS Trust<br>Green, Jennifer; North West Anglia NHS Foundation Trust, Department of Obstetrics & Gynaecology<br>Vaninetti, Matilde; Cambridge University Hospitals NHS Foundation Trust<br>Shah, Nishel; Chelsea and Westminster Hospital, Department of Metabolism, Digestion and Reproduction; Chelsea and Westminster Hospital NHS Foundation Trust<br>Greer, Orene; Chelsea and Westminster Hospital, Department of Metabolism, Digestion and Reproduction; Chelsea and Westminster Hospital NHS Foundation Trust
**Primary Subject Heading**:	Obstetrics and gynaecology
Secondary Subject Heading:	Diagnostics, Ethics, Obstetrics and gynaecology, Qualitative research
Keywords:	OBSTETRICS, GYNAECOLOGY, Systematic Review, Meta-Analysis

SCHOLARONE™
Manuscripts

The use of artificial intelligence in obstetric and gynaecological diagnostics: a protocol for a systematic review and meta-analysis

Anjalee Chaurasia[1], Georgia Curry[1], Yi Zhao[1], Fatema Dawoodbhoy[2], Jennifer Green[3], Matilde Vaninetti[4], Nishel Shah[5,6], Orene Greer[5,6]

Anjalee Chaurasia and Georgia Curry are the joint first authors*

[1] Imperial College London School of Medicine, London, United Kingdom

[2] Barking, Havering and Redbridge NHS Trust, United Kingdom

[3] Department of Obstetrics & Gynaecology, Hinchingbrooke Hospital, Northwest Anglia NHS Foundation Trust, United Kingdom.

[4] Cambridge University Hospitals NHS Foundation Trust, United Kingdom

[5] Imperial College London, Department of Metabolism, Digestion and Reproduction, Chelsea and Westminster Hospital, London, United Kingdom.

[6] Chelsea & Westminster NHS Foundation Trust, 369 Fulham Road, London, United Kingdom

Correspondence to: Anjalee Chaurasia

For peer review only

Imperial College London, Faculty of Medicine

Ayrton Rd, South Kensington, London SW7 5NH

aac3018@ic.ac.uk;

Ethics: No ethical considerations apply.

Word count: 1874

For peer review only

ABSTRACT

Introduction: Emerging developments in applications of artificial intelligence (AI) in healthcare offer the opportunity to improve diagnostic capabilities in obstetrics and gynaecology (O&G), ensuring early detection of pathology, optimal management and improving survival. Consensus on a robust AI healthcare framework is crucial for standardizing protocols that promote data privacy and transparency, minimize bias, and ensure patient safety. Here, we describe the study protocol for a systematic review and meta-analysis to evaluate current applications of AI in O&G diagnostics with consideration of reporting standards used and their ethical implications. This protocol is written following the Preferred Reporting Items for Systematic Review and Meta-Analysis Protocols (PRISMA-P) 2015 checklist.

Methods and analysis: The study objective is to explore the current application of AI in O&G diagnostics and assess the reporting standards used in these studies. Electronic bibliographic databases MEDLINE, EMBASE and Cochrane will be searched. Study selection, data extraction, and subsequent narrative synthesis and meta-analyses will be carried out following the PRISMA-P guidelines. Included

For peer review only - http://bmjopen.bmj.com/site/about/guidelines.xhtml

papers will be English-language full-text articles from May 2015 to March 2024 which provide original data, as AI has been redefined in recent literature. Papers must use AI as the predictive method, focusing on improving O&G diagnostic outcomes.

We will evaluate the reporting standards including the risk of bias, lack of transparency and consider the ethical implications and potential harm to patients. Outcome measures will involve assessing the included studies against gold standard criteria for robustness of model development (Transparent reporting of a multivariable prediction model for individual prognosis or diagnosis (TRIPOD), model predictive performance, model risk of bias and applicability (Prediction model Risk Of Bias Assessment Tool (PROBAST) and study reporting (Consolidated Standards of Reporting Trials-Artificial Intelligence) CONSORT-AI guidance.

Ethics and dissemination: Ethical approval is not required for this systematic review. Findings will be shared through peer-reviewed publications. There will be no patient or public involvement in this study.

PROSPERO registration number: CRD42022357024

Strengths and limitations of the study:

- To our knowledge, this will be the first comprehensive systematic review and meta-analysis of the ethical considerations of the application of AI in O&G diagnostics.

For peer review only

- Statistical analysis will be performed to determine the effect of heterogeneity in the literature.

- Heterogeneity of study methods may impact the strength of the conclusions.

- The analysis may be limited if a small study number meets the inclusion criteria.

For peer review only

BACKGROUND

Access to accurate diagnostics is fundamental to reducing morbidity and mortality. However, common diagnostic tools used in areas of obstetrics and gynaecology (O&G) have their limitations. Many of the diagnostics are performed using ultrasound, which is subjective and prone to error [1,2]. For instance, inter and intra-operator variation in foetal birth weight estimates and discrepancies in nuchal translucency are acknowledged [3–5]. Another example is in the tools used to predict preterm birth, a major cause of neonatal death worldwide. Additional work is required to improve our ability to accurately predict it and subsequently improve clinical outcomes [6,7]; particularly as much of the resultant mortality might be prevented with cost-effective interventions [8–10]. Thus, emerging technological AI developments in medicine may offer an opportunity to improve diagnostic capabilities in O&G [11].

Artificial intelligence (AI) uses multifactorial data to build connections and learn. Such data can take the form of images or numeric values obtained from existing databases or laboratory tests [12]. For example, machine learning models can be applied to a series of medical images to extract key features associated with specific pathologies and this can be used to increase the accuracy of detecting

For peer review only

gynaecological malignancy e.g., malignant epithelial ovarian tumours [13]. It has been suggested that AI has the potential to perform problem-solving at a greater efficiency than humans [14].

However, the development in AI technology requires parallel development of rigorous protocols, which can be applied across AI applications, to ensure data privacy, transparency, safety and fairness, as well as to minimise bias. The importance of improved governance of AI research can be demonstrated by the example of an algorithm which was developed to determine patient health risks for approximately 200 million individuals in the US each year [15]. It was shown to exhibit racial bias, with the choice of label used to train this data resulting in fewer black patients receiving the care they needed [15].

The implementation of this new technology, if unregulated, has the potential to cause personal and societal harm, with risk to patient safety [16]. Foremost, AI algorithms may be subject to predictive error due to the training data set used. This may result in incorrect clinical findings and subsequent under or over treatment, unknown to both clinician and patient [17]. Therefore, it is important that researchers developing AI models acknowledge the ethical implications AI may pose for healthcare and adhere to recommended standards for reporting of their methods and data. We aim to evaluate current practice and highlight where work is required to improve this. Thus, we will systematically review existing studies, assessing reporting standards, performance, model development and performance, and adherence to 'gold standard' criteria.

METHODS AND ANALYSIS

For peer review only

The protocol is registered with the international Prospective Register of Systematic Reviews (PROSPERO), registration number CRD42022357024. The protocol and the completed systematic review will be reported using the Preferred Reporting Items for Systematic Reviews and Meta-analyses for protocols (PRISMA-P) [18].

Search Methodology

The search will be carried out using Medical Literature Analysis and Retrieval System Online (MEDLINE), Excerpta Medica Database (EMBASE) and Cochrane. The search strategy will include Medical Subject Headings (MESH) terms, free text and Boolean operators. We will search using the terms “obstetrics” and “gynaecology”, multiple synonyms of “artificial intelligence” including “machine learning”, “deep learning” and “algorithm”, along with “diagnostics”, “prognostics” and their variations. The search strategy will be adapted to the specific database. Full search strategy can be found in the supplementary file. A manual search of the grey literature will also be performed. The studies included will only be in the English language, published from May 2015 to March 2024, as AI methods was recently redefined in a seminal article published in 2015 [19].

Study selection and data extraction

For peer review only

The studies will be uploaded to a systematic review study management software, for example Rayyan [20], to remove duplicates. The studies will be screened by 2 independent reviewers, comparing the titles and abstracts to the inclusion criteria. The same process will then be carried out for the full text of the selected studies. Any disagreements between the reviewers will be resolved via discussion or following discussion with an additional reviewer. Study exclusion criteria will be recorded and reported in the review. The references of manuscripts identified from the search will also be screened for studies suitable for inclusion. A PRISMA flow chart of the search and study selection will be included in the report [21].

Inclusion criteria

This systematic review will include peer-reviewed studies that use patient data for predictive outcomes. To be included in the analysis, studies must use AI as the predictive method, focusing on improving O&G diagnostic outcomes and be written in English.

Exclusion criteria

Non-human studies and papers that do not use patient data for predictive outcomes. Review articles, commentaries, abstracts, meta-analyses, case reports, and expert opinions, will be excluded. Literature

For peer review only

that does not discuss AI in O&G diagnostics and papers not published in the English language will also be excluded.

Data extraction

Data will be extracted from the studies using Excel. The data extracted will include the Year of publication, Study authors, Study design, Patient population, Study size, the O&G pathology evaluated, the diagnostic technique, type of AI model, reporting system for example TRIPOD-AI, the performance measurements used, the method of clinical deployment of the AI model and whether ethical approval was granted for each study. In cases of incomplete or missing data in published studies, the authors will be contacted to capture the full data for extraction. Table 1 summarises the data to be extracted.

For peer review on

Table 1 Data collection items

**Item no.**	Data title	Data type
**1**	Year of publication	Study characteristic
**2**	Study authors	Study characteristic
**3**	Study design	Study characteristic

4	Patient population	Demographics
5	Study size	Demographics
6	Target O&G pathology	Methodology
7	Diagnostic technique	Methodology
8	Type of AI model	Outcome
9	Reporting system used	Outcome
10	Method of illustrating diagnostic performance	Outcome
11	Method of model deployment	Outcome
12	Whether ethical approval was granted for each study (Y/N)	Outcome

148 *O&G- Obstetrics and gynaecology. AI- artificial intelligence.*

149 **Endpoints**

150 The primary endpoint is a demonstration of the application of current recommended standards for the 151 development and reporting of AI primary research involving O&G diagnostics (relative to CONSORT-152 AI, Transparent reporting of a multivariable prediction model for individual prognosis or diagnosis

For peer review only - http://bmjopen.bmj.com/site/about/guidelines.xhtml

(TRIPOD) (22) and Prediction model Risk Of Bias Assessment Tool (PROBAST) guidelines) (23). We will summarise these findings in a table.

Meta-analysis

If there are sufficient papers illustrating the diagnostic performance of the AI models for diseases relating to the field of obstetrics and gynaecology, a meta-analysis will be conducted. Model performance measures, including sensitivity, specificity and the area under the receiver operating curve (AUROC) values for specific O&G endpoints will be compared in the meta-analysis. The pooled sensitivity and specificity can be compared to the reference standards and reported. If any of the required data has not been reported, we will contact the study authors to request data sharing. If studies present a different metric for model performance, these will also be included in the report.

A 2x2 contingency table containing true positive, false positive, true negative, false negative will be created for the sensitivity and specificity of each study and compared using bivariate analysis. These will subsequently be plotted on forest plots and summary receiver operating characteristic (sROC) curve plots. The sROC will be calculated with the individual AUC.

Interstudy variation and heterogeneity will be displayed using I^2. To obtain pooled estimates, we will use a random-effects model. Leave-one-out analyses will be used to detect outliers, and the model will be changed accordingly. To control for study variance, we will use an untransformed, logit and double-

For peer review only

arcsine transformed proportions, and normality will be assessed using the Shapiro-Wilk test and density plots. Ratios resembling normal distributions will be used. Analysis and visualization will be carried out using R statistical environment (V.3.6.1, 2019-07-05).

Risk of bias in individual studies

PROBAST will be used to assess the included studies for bias and applicability [22]. It is divided into 4 domains: participants, predictors, outcome, and analysis. It has 20 questions that help to judge bias. Bias occurs when the shortcomings in the study design, conduct or analysis impact predictive performance. It will give an idea of the reliability of the data produced and the appropriateness of the model for diagnosis and prognosis. If studies with high bias are included, the impact and reasons for this will be discussed.

Patient and Public involvement

There will be no patient or public involvement in this study.

Trial status

- Preliminary searches: started

For peer review only

- Piloting of the study selection process: started
- Formal screening: not started
- Data extraction: not started
- Risk of bias assessment: not started
- Data analysis: not started

ETHICS AND DISSEMINATION

There are no ethical concerns as the review does not require consent or subject participation. Dissemination of the protocol and systematic review will be done through a peer-reviewed journal.

Author Affiliations

[1] Imperial College London School of Medicine, London, UK

[2] Barking, Havering and Redbridge NHS Trust

For peer review only

[3] Department of Obstetrics & Gynaecology, Hinchingbrooke Hospital, Northwest Anglia NHS Foundation Trust, UK.

[4] Imperial College London, Department of Metabolism, Digestion and Reproduction, Chelsea and Westminster Hospital, London, United Kingdom.

[5] Cambridge University Hospitals NHS Foundation Trust

Email: Anjalee Chaurasia: aac3018@ic.ac.uk, Georgia Curry: glc17@ic.ac.uk, Yi Zhao: yi.zhao18@ic.ac.uk

Contributors: The authors' contribution includes, but is not limited to, the following: GC, AC and YZ drafted the manuscript and created the study concept. OG, NS, FD, JG and MV provided supervision and guidance during the study. All authors reviewed and approved the manuscript in its current form. GC and AC are the guarantors of this work.

Funding: All authors acknowledge the funding from the Imperial College London Open Access Fund

Acknowledgements: We would like to acknowledge and thank Mr Phillip Barlow who assisted with optimising and refining our proposed search strategy. Citation: Evidence search: Machine learning for use in diagnostic tools for obstetrics & gynaecology. Phillip Barlow (Imperial College Library Services). (6th March, 2024). LONDON, UK: Imperial College NHS and Chelsea & Westminster Hospital NHS Trusts Libraries.

For peer review only

Competing Interests: None declared.

Patient consent for publication: Not required

Provenance and peer review: Not commissioned; externally peer-reviewed

Open access This is an open-access article distributed in accordance with the Creative Commons Attribution 4.0 Unreported (CC BY 4.0) license which permits others to copy, redistribute, remix, transform and build upon this work for any purpose, provided the original work is properly cited, a link to the license is given, and indication of whether changes were made. See https://creativecommons.org/licenses/by/4.0/.

ORCHID IDs

- Anjalee Chaurasia https://orchid.org/0000-0003-3924-9201
- Georgia Curry https://orcid.org/0000-0002-2888-3859
- Yi Zhao https://orcid.org/0000-0002-4563-4344
- Fatema Dawoodbhoy https://orchid.org/0000-0002-7255-688X
- Nishel Shah https://orcid.org/0000-0002-5694-9915
- Orene Greer https://orcid.org/0000-0002-4940-0828

For peer review only

REFERENCES

1 Sotiriadis A, Odibo AO. Systematic error and cognitive bias in obstetric ultrasound. *Ultrasound Obstet Gynecol*. 2019;53:431–5.

2 Dudley NJ. The management of error in ultrasound fetal growth monitoring. *Ultrasound*. 2021;29:4–9.

3 Chen Z, Liu Z, Du M, *et al.* Artificial Intelligence in Obstetric Ultrasound: An Update and Future Applications. *Front Med (Lausanne)*. 2021;8:733468.

4 Milner J, Arezina J. The accuracy of ultrasound estimation of fetal weight in comparison to birth weight: A systematic review. *Ultrasound*. 2018;26:32–41.

5 Benacerraf BR, Minton KK, Benson CB, *et al.* Proceedings: Beyond Ultrasound First Forum on improving the quality of ultrasound imaging in obstetrics and gynecology. *Am J Obstet Gynecol*. 2018;218:19–28.

For peer review only

6 Stock SJ, Horne M, Bruijn M, *et al.* Development and validation of a risk prediction model of preterm birth for women with preterm labour symptoms (the QUIDS study): A prospective cohort study and individual participant data meta-analysis. *PLoS Med*. 2021;18:e1003686.

7 Suff N, Story L, Shennan A. The prediction of preterm delivery: What is new? *Semin Fetal Neonatal Med*. 2019;24:27–32.

8 Lee K-S, Ahn KH. Application of Artificial Intelligence in Early Diagnosis of Spontaneous Preterm Labor and Birth. *Diagnostics (Basel)*. 2020;10:733.

9 Iftikhar P, Kuijpers MV, Khayyat A, *et al.* Artificial Intelligence: A New Paradigm in Obstetrics and Gynecology Research and Clinical Practice. *Cureus*. 2020;12:e7124.

10 Walani SR. Global burden of preterm birth. *Int J Gynaecol Obstet*. 2020;150:31–3.

11 Kim HY, Cho GJ, Kwon HS. Applications of artificial intelligence in obstetrics. *Ultrasonography*. 2023;42:2–9.

12 O'Leary DE. Artificial Intelligence and Big Data. *IEEE Intelligent Systems*. 2013;28:96–9.

13 Li Y, Jian J, Pickhardt PJ, *et al.* MRI-Based Machine Learning for Differentiating Borderline From Malignant Epithelial Ovarian Tumors: A Multicenter Study. *J Magn Reson Imaging*. 2020;52:897–904.

14 Davenport T, Kalakota R. The potential for artificial intelligence in healthcare. *Future Healthc J*. 2019;6:94–8.

15 Obermeyer Z, Powers B, Vogeli C, *et al.* Dissecting racial bias in an algorithm used to manage the health of populations. *Science*. 2019;366:447–53.

16 Kim DW, Jang HY, Kim KW, *et al.* Design Characteristics of Studies Reporting the Performance of Artificial Intelligence Algorithms for Diagnostic Analysis of Medical Images: Results from Recently Published Papers. *Korean J Radiol*. 2019;20:405–10.

17 Kristiansen TB, Kristensen K, Uffelmann J, *et al.* Erroneous data: The Achilles' heel of AI and personalized medicine. *Front Digit Health*. 2022;4:862095.

18 Moher D, Shamseer L, Clarke M, *et al.* Preferred reporting items for systematic review and meta-analysis protocols (PRISMA-P) 2015 statement. *Systematic Reviews*. 2015;4:1.

19 LeCun Y, Bengio Y, Hinton G. Deep learning. *Nature*. 2015;521:436–44.

20 Ouzzani M, Hammady H, Fedorowicz Z, *et al.* Rayyan-a web and mobile app for systematic reviews. *Syst Rev*. 2016;5:210.

For peer review only

288 21 Page MJ, McKenzie JE, Bossuyt PM, *et al.* The PRISMA 2020 statement: an updated guideline for reporting systematic reviews. *BMJ*. 2021;372:n71.

290 22 Wolff RF, Moons KGM, Riley RD, *et al.* PROBAST: A Tool to Assess the Risk of Bias and Applicability of Prediction Model Studies. *Ann Intern Med*. 2019;170:51–8.

292

For peer review only

The use of artificial intelligence in obstetric and gynaecological diagnostics: a protocol for a systematic review and meta-analysis

The full search strategy to this systematic review are as follows:

Date range used (5 years, 10 years): May 2015 to date
Limits used (gender, article/study type, etc.): Female, human
Search terms and notes (full search strategy for database searches below):

Keyword terms

- Artificial Intelligence
  - machine learning or supervised learning or weak supervision or semi-supervised learning or active learning or structured prediction
  - structured learning or output learning or "learning to rank" or machine-learned ranking or unsupervised learning or reinforcement learning
  - neural network or artificial neural network or neural net
  - artificial intelligence or AI or deep learning
- Obstetrics and Gynaecology
  - "obstetrics and gynaecology" or "obstetrics and gynecology" or obstet* or gyn?ecol*
  - maternal-f?etal medicine or perinatology
  - "Reproductive endocrinology and infertility" or reproductive surgery
  - Gyn?ecological oncology or urogyn?ecology
  - Gyn?ecological oncology or urogyn?ecology or "female pelvic medicine and reconstructive surgery"
  - family planning or abortion or induced miscarriage or "termination of pregnancy" or (pregnanc* adj3 terminat*) or birth control or contracep*
- Diagnostic Imaging
  - medical imaging or diagnostic imaging
  - radiography or x-ray or fluoroscop* or projectional radiography or conventional radiography or plain radiography or roentgenography
  - magnetic resonance imaging or MRI
  - nuclear medicine or nucleology
  - medical ultrasound or medical ultrasonography or sonography or echography or ultrasound
  - computed tomography or CT Scan

Subject headings

	MEDLINE	Embase	Cochrane
Artificial Intelligence	Artificial Intelligence	Machine Learning	Artificial Intelligence
	Machine Learning	Semi Supervised Machine Learning	Machine Learning
	Deep Learning	Supervised Machine Learning	Deep Learning
	Supervised Machine Learning	Support Vector Machine	Supervised Machine Learning

For peer review only

	Support Vector Machine	Unsupervised Machine Learning	Support Vector Machine
	Unsupervised Machine Learning	Deep Learning	Unsupervised Machine Learning
	Sentiment Analysis	Artificial Intelligence	Sentiment Analysis
Obstetrics and Gynaecology	Gynecology	Gynecology	Gynecology
	Obstetrics	Obstetrics	Obstetrics
	Women's Health	Women's Health	Women's Health
	Maternal Health	Maternal Care	Maternal Health
	Reproductive Medicine	Reproduction	Reproductive Medicine
	Gynecology	Childbirth	Gynecology
	Reproductive Health	Pregnancy	Reproductive Health
	Sexual Health	Prenatal Development	Sexual Health
		Pseudopregnancy	
		Puerperium	
		Reproductive Health	
		Sexual Health	
Diagnostic Imaging	Diagnostic Imaging (exp)	Diagnostic Imaging (exp)	Diagnostic Imaging (exp)
		Computer Assisted Tomography	
		Radiography	
		Nuclear Magnetic Resonance Imaging (exp)	
		Nuclear Medicine	
		Echography	

The search strategy was devised and subsequently run in three resources across two platforms:

- Ovid
  - MEDLINE
  - Embase
- The Cochrane Library
-

Search History

Ovid MEDLINE(R) ALL <May 2015 to March 04, 2024>

1 (machine learning or supervised learning or weak supervision or semi-supervised learning or active learning or structured prediction).mp. [mp=title, book title, abstract, original title, name of substance word, subject heading word, floating sub-heading word, keyword heading word, organism supplementary concept word, protocol supplementary concept word, rare disease supplementary concept word, unique identifier, synonyms, population supplementary concept word, anatomy supplementary concept word]

For peer review only

2 (structured learning or output learning or "learning to rank" or machine-learned ranking or unsupervised learning or reinforcement learning).mp. [mp=title, book title, abstract, original title, name of substance word, subject heading word, floating sub-heading word, keyword heading word, organism supplementary concept word, protocol supplementary concept word, rare disease supplementary concept word, unique identifier, synonyms, population supplementary concept word, anatomy supplementary concept word]

3 (neural network or artificial neural network or neural net).mp. [mp=title, book title, abstract, original title, name of substance word, subject heading word, floating sub-heading word, keyword heading word, organism supplementary concept word, protocol supplementary concept word, rare disease supplementary concept word, unique identifier, synonyms, population supplementary concept word, anatomy supplementary concept word]

4 (artificial intelligence or AI or deep learning).mp. [mp=title, book title, abstract, original title, name of substance word, subject heading word, floating sub-heading word, keyword heading word, organism supplementary concept word, protocol supplementary concept word, rare disease supplementary concept word, unique identifier, synonyms, population supplementary concept word, anatomy supplementary concept word]

5 artificial intelligence/ or machine learning/ or deep learning/ or supervised machine learning/ or support vector machine/ or unsupervised machine learning/ or sentiment analysis/

6 1 or 2 or 3 or 4 or 5

7 ("obstetrics and gynaecology" or "obstetrics and gynecology" or obstet* or gyn?ecol*).mp. [mp=title, book title, abstract, original title, name of substance word, subject heading word, floating sub-heading word, keyword heading word, organism supplementary concept word, protocol supplementary concept word, rare disease supplementary concept word, unique identifier, synonyms, population supplementary concept word, anatomy supplementary concept word]

8 (maternal-f?etal medicine or perinatology).mp. [mp=title, book title, abstract, original title, name of substance word, subject heading word, floating sub-heading word, keyword heading word, organism supplementary concept word, protocol supplementary concept word, rare disease supplementary concept word, unique identifier, synonyms, population supplementary concept word, anatomy supplementary concept word]

9 ("Reproductive endocrinology and infertility" or reproductive surgery).mp. [mp=title, book title, abstract, original title, name of substance word, subject heading word, floating sub-heading word, keyword heading word, organism supplementary concept word, protocol supplementary concept word, rare disease supplementary concept word, unique identifier, synonyms, population supplementary concept word, anatomy supplementary concept word]

10 (Gyn?ecological oncology or urogyn?ecology).mp. [mp=title, book title, abstract, original title, name of substance word, subject heading word, floating sub-heading word, keyword heading word, organism supplementary concept word, protocol supplementary concept word, rare disease supplementary concept word, unique identifier, synonyms, population supplementary concept word, anatomy supplementary concept word]

For peer review only

11 (Gyn?ecological oncology or urogyn?ecology or "female pelvic medicine and reconstructive surgery").mp. [mp=title, book title, abstract, original title, name of substance word, subject heading word, floating sub-heading word, keyword heading word, organism supplementary concept word, protocol supplementary concept word, rare disease supplementary concept word, unique identifier, synonyms, population supplementary concept word, anatomy supplementary concept word]

12 (family planning or abortion or induced miscarriage or "termination of pregnancy" or (pregnanc* adj3 terminat*) or birth control or contracep*).mp. [mp=title, book title, abstract, original title, name of substance word, subject heading word, floating sub-heading word, keyword heading word, organism supplementary concept word, protocol supplementary concept word, rare disease supplementary concept word, unique identifier, synonyms, population supplementary concept word, anatomy supplementary concept word]

13 gynecology/ or obstetrics/

14 women's health/ or maternal health/

15 reproductive medicine/ or gynecology/

16 reproductive health/ or sexual health/

17 7 or 8 or 9 or 10 or 11 or 12 or 13 or 14 or 15 or 16

18 (medical imaging or diagnostic imaging).mp. [mp=title, book title, abstract, original title, name of substance word, subject heading word, floating sub-heading word, keyword heading word, organism supplementary concept word, protocol supplementary concept word, rare disease supplementary concept word, unique identifier, synonyms, population supplementary concept word, anatomy supplementary concept word]

19 (radiography or x-ray or flouroscop* or projectional radiography or conventional radiography or plain radiography or roentgenography).mp. [mp=title, book title, abstract, original title, name of substance word, subject heading word, floating sub-heading word, keyword heading word, organism supplementary concept word, protocol supplementary concept word, rare disease supplementary concept word, unique identifier, synonyms, population supplementary concept word, anatomy supplementary concept word]

20 (magnetic resonance imaging or MRI).mp. [mp=title, book title, abstract, original title, name of substance word, subject heading word, floating sub-heading word, keyword heading word, organism supplementary concept word, protocol supplementary concept word, rare disease supplementary concept word, unique identifier, synonyms, population supplementary concept word, anatomy supplementary concept word]

21 (nuclear medicine or nucleology).mp. [mp=title, book title, abstract, original title, name of substance word, subject heading word, floating sub-heading word, keyword heading word, organism supplementary concept word, protocol supplementary concept word, rare disease supplementary concept word, unique identifier, synonyms, population supplementary concept word, anatomy supplementary concept word]

For peer review only

22 (medical ultrasound or medical ultrasonography or sonography or echography or ultrasound).mp. [mp=title, book title, abstract, original title, name of substance word, subject heading word, floating sub-heading word, keyword heading word, organism supplementary concept word, protocol supplementary concept word, rare disease supplementary concept word, unique identifier, synonyms, population supplementary concept word, anatomy supplementary concept word]

23 (computed tomography or CT Scan).mp. [mp=title, book title, abstract, original title, name of substance word, subject heading word, floating sub-heading word, keyword heading word, organism supplementary concept word, protocol supplementary concept word, rare disease supplementary concept word, unique identifier, synonyms, population supplementary concept word, anatomy supplementary concept word]

24 exp Diagnostic Imaging/

25 18 or 19 or 20 or 21 or 22 or 23 or 24

26 6 and 17 and 25

27 (Animals/ not (Animals/ and Humans/)) or (exp animals/ not humans.sh.)

28 26 not 27

29 limit 28 to yr="2015 -Current"

Embase <May 2015 to 2024 March 04>

1 (machine learning or supervised learning or weak supervision or semi-supervised learning or active learning or structured prediction).mp. [mp=title, abstract, heading word, drug trade name, original title, device manufacturer, drug manufacturer, device trade name, keyword heading word, floating subheading word, candidate term word]

2 (structured learning or output learning or "learning to rank" or machine-learned ranking or unsupervised learning or reinforcement learning).mp. [mp=title, abstract, heading word, drug trade name, original title, device manufacturer, drug manufacturer, device trade name, keyword heading word, floating subheading word, candidate term word]

3 (neural network or artificial neural network or neural net).mp. [mp=title, abstract, heading word, drug trade name, original title, device manufacturer, drug manufacturer, device trade name, keyword heading word, floating subheading word, candidate term word]

4 (artificial intelligence or AI or deep learning).mp. [mp=title, abstract, heading word, drug trade name, original title, device manufacturer, drug manufacturer, device trade name, keyword heading word, floating subheading word, candidate term word]

5 machine learning/ or semi supervised machine learning/ or supervised machine learning/ or support vector machine/ or unsupervised machine learning/

6 deep learning/

For peer review only

7 artificial intelligence/

8 1 or 2 or 3 or 4 or 5 or 6 or 7

9 ("obstetrics and gynaecology" or "obstetrics and gynecology" or obstet* or gyn?ecol*).mp. [mp=title, abstract, heading word, drug trade name, original title, device manufacturer, drug manufacturer, device trade name, keyword heading word, floating subheading word, candidate term word]

10 (maternal-f?etal medicine or perinatology).mp. [mp=title, abstract, heading word, drug trade name, original title, device manufacturer, drug manufacturer, device trade name, keyword heading word, floating subheading word, candidate term word]

11 ("Reproductive endocrinology and infertility" or reproductive surgery).mp. [mp=title, abstract, heading word, drug trade name, original title, device manufacturer, drug manufacturer, device trade name, keyword heading word, floating subheading word, candidate term word]

12 (Gyn?ecological oncology or urogyn?ecology).mp. [mp=title, abstract, heading word, drug trade name, original title, device manufacturer, drug manufacturer, device trade name, keyword heading word, floating subheading word, candidate term word]

13 (Gyn?ecological oncology or urogyn?ecology or "female pelvic medicine and reconstructive surgery").mp. [mp=title, abstract, heading word, drug trade name, original title, device manufacturer, drug manufacturer, device trade name, keyword heading word, floating subheading word, candidate term word]

14 (family planning or abortion or induced miscarriage or "termination of pregnancy" or (pregnanc* adj3 terminat*) or birth control or contracep*).mp. [mp=title, abstract, heading word, drug trade name, original title, device manufacturer, drug manufacturer, device trade name, keyword heading word, floating subheading word, candidate term word]

15 gynecology/ or obstetrics/

16 women's health/

17 exp maternal care/

18 reproduction/ or childbirth/ or pregnancy/ or prenatal development/ or pseudopregnancy/ or puerperium/

19 reproductive health/

20 sexual health/

21 9 or 10 or 11 or 12 or 13 or 14 or 15 or 16 or 17 or 18 or 19 or 20

22 (medical imaging or diagnostic imaging).mp. [mp=title, abstract, heading word, drug trade name, original title, device manufacturer, drug manufacturer, device trade name, keyword heading word, floating subheading word, candidate term word]

For peer review only

23 (radiography or x-ray or flouroscop* or projectional radiography or conventional radiography or plain radiography or roentgenography).mp. [mp=title, abstract, heading word, drug trade name, original title, device manufacturer, drug manufacturer, device trade name, keyword heading word, floating subheading word, candidate term word]

24 (magnetic resonance imaging or MRI).mp. [mp=title, abstract, heading word, drug trade name, original title, device manufacturer, drug manufacturer, device trade name, keyword heading word, floating subheading word, candidate term word]

25 (nuclear medicine or nucleology).mp. [mp=title, abstract, heading word, drug trade name, original title, device manufacturer, drug manufacturer, device trade name, keyword heading word, floating subheading word, candidate term word]

26 (medical ultrasound or medical ultrasonography or sonography or echography or ultrasound).mp. [mp=title, abstract, heading word, drug trade name, original title, device manufacturer, drug manufacturer, device trade name, keyword heading word, floating subheading word, candidate term word]

27 (computed tomography or CT Scan).mp. [mp=title, abstract, heading word, drug trade name, original title, device manufacturer, drug manufacturer, device trade name, keyword heading word, floating subheading word, candidate term word]

28 exp diagnostic imaging/

29 exp computer assisted tomography/

30 radiography/

31 exp nuclear magnetic resonance imaging/

32 nuclear medicine/

33 exp echography/

34 22 or 23 or 24 or 25 or 26 or 27 or 28 or 29 or 30 or 31 or 32 or 33

35 8 and 21 and 34

36 (exp animal/ or nonhuman/) not exp human/

37 35 not 36

38 limit 37 to yr="2015 -Current"

Cochrane

Search

For peer review only

#1 machine learning or supervised learning or weak supervision or semi-supervised learning or active learning or structured prediction

#2 structured learning or output learning or "learning to rank" or machine-learned ranking or unsupervised learning or reinforcement learning

#3 neural network or artificial neural network or neural net

#4 artificial intelligence or AI or deep learning

#5 MeSH descriptor: [Artificial Intelligence] this term only

#6 MeSH descriptor: [Machine Learning] this term only

#7 MeSH descriptor: [Deep Learning] this term only

#8 MeSH descriptor: [Supervised Machine Learning] this term only

#9 MeSH descriptor: [Support Vector Machine] this term only

#10 MeSH descriptor: [Unsupervised Machine Learning] this term only

#11 MeSH descriptor: [Sentiment Analysis] explode all trees

#12 #1 OR #2 OR #3 OR #4 OR #5 OR #6 OR #7 OR #8 OR #9 OR #10 OR #11

#13 "obstetrics and gynaecology" or "obstetrics and gynecology" or obstet* or gyn?ecol*

#14 maternal-f?etal medicine or perinatology

#15 "Reproductive endocrinology and infertility" or reproductive surgery

#16 Gyn?ecological oncology or urogyn?ecology

#17 Gyn?ecological oncology or urogyn?ecology or "female pelvic medicine and reconstructive surgery"

#18 family planning or abortion or induced miscarriage or "termination of pregnancy" or (pregnanc* NEAR/3 terminat*)

#19 MeSH descriptor: [Gynecology] this term only

#20 MeSH descriptor: [Obstetrics] this term only

#21 MeSH descriptor: [Women's Health] this term only

#22 MeSH descriptor: [Maternal Health] this term only

For peer review only

#23 MeSH descriptor: [Reproductive Medicine] this term only

#24 MeSH descriptor: [Reproductive Health] this term only

#25 MeSH descriptor: [Sexual Health] this term only

#26 #13 OR #14 OR #15 OR #16 OR #17 OR #18 OR #19 OR #20 OR #21 OR #22 OR #23 OR #24 OR #25

#27 medical imaging or diagnostic imaging

#28 radiography or x-ray or flouroscop* or projectional radiography or conventional radiography or plain radiography or roentgenography

#29 magnetic resonance imaging or MRI

#30 nuclear medicine or nucleology

#31 medical ultrasound or medical ultrasonography or sonography or echography or ultrasound

#32 computed tomography or CT Scan

#33 MeSH descriptor: [Diagnostic Imaging] explode all trees

#34 #27 OR #28 OR #29 OR #30 OR #31 OR #32 OR #33

#35 #12 AND #26 AND #34

#36 Limit to custom date range 2015-2024 149 Cochrane Reviews / 16 Cochrane Protocols / 76 Cochrane Central Register Trials

For peer review only

PRISMA-P 2015 Checklist

This checklist has been adapted for use with protocol submissions to *Systematic Reviews* from Table 3 in Moher D et al: Preferred reporting items for systematic review and meta-analysis protocols (PRISMA-P) 2015 statement. *Systematic Reviews* 2015 **4**:1

Section/topic	#	Checklist item	Information reported Yes	Information reported No	Line number(s)
**ADMINISTRATIVE INFORMATION**					
**Title**					
Identification	1a	Identify the report as a protocol of a systematic review	☒	☐	1
Update	1b	If the protocol is for an update of a previous systematic review, identify as such	☐	☒	
**Registration**	2	If registered, provide the name of the registry (e.g., PROSPERO) and registration number in the Abstract	☒	☐	53
**Authors**					
Contact	3a	Provide name, institutional affiliation, and e-mail address of all protocol authors; provide physical mailing address of corresponding author	☒	☐	4-20
Contributions	3b	Describe contributions of protocol authors and identify the guarantor of the review	☒	☐	220-223
**Amendments**	4	If the protocol represents an amendment of a previously completed or published protocol, identify as such and list changes; otherwise, state plan for documenting important protocol amendments	☒	☐	228-232
**Support**					
Sources	5a	Indicate sources of financial or other support for the review	☒	☐	224
Sponsor	5b	Provide name for the review funder and/or sponsor	☒	☐	224
Role of sponsor/funder	5c	Describe roles of funder(s), sponsor(s), and/or institution(s), if any, in developing the protocol	☒	☐	224
**INTRODUCTION**					
**Rationale**	6	Describe the rationale for the review in the context of what is already known	☒	☐	70-90
**Objectives**	7	Provide an explicit statement of the question(s) the review will address with reference to participants, interventions, comparators, and outcomes (PICO)	☒	☐	36-49
**METHODS**					

For peer review only

BioMed Central The Open Access Publisher

Section/topic	#	Checklist item	Information reported Yes	Information reported No	Line number(s)
**Eligibility criteria**	8	Specify the study characteristics (e.g., PICO, study design, setting, time frame) and report characteristics (e.g., years considered, language, publication status) to be used as criteria for eligibility for the review	☒	☐	131-133
**Information sources**	9	Describe all intended information sources (e.g., electronic databases, contact with study authors, trial registers, or other grey literature sources) with planned dates of coverage	☒	☐	109-110
**Search strategy**	10	Present draft of search strategy to be used for at least one electronic database, including planned limits, such that it could be repeated	☒	☐	198-202
STUDY RECORDS					
Data management	11a	Describe the mechanism(s) that will be used to manage records and data throughout the review	☒	☐	153-173
Selection process	11b	State the process that will be used for selecting studies (e.g., two independent reviewers) through each phase of the review (i.e., screening, eligibility, and inclusion in meta-analysis)	☒	☐	121-128
Data collection process	11c	Describe planned method of extracting data from reports (e.g., piloting forms, done independently, in duplicate), any processes for obtaining and confirming data from investigators	☒	☐	121-128
**Data items**	12	List and define all variables for which data will be sought (e.g., PICO items, funding sources), any pre-planned data assumptions and simplifications	☒	☐	142-148
**Outcomes and prioritization**	13	List and define all outcomes for which data will be sought, including prioritization of main and additional outcomes, with rationale	☒	☐	142-148
**Risk of bias in individual studies**	14	Describe anticipated methods for assessing risk of bias of individual studies, including whether this will be done at the outcome or study level, or both; state how this information will be used in data synthesis	☒	☐	176-181
DATA					
**Synthesis**	15a	Describe criteria under which study data will be quantitatively synthesized	☒	☐	157-163
	15b	If data are appropriate for quantitative synthesis, describe planned summary measures, methods of handling data, and methods of combining data from studies, including any planned exploration of consistency (e.g., I^2, Kendall's tau)	☒	☐	164-173
	15c	Describe any proposed additional analyses (e.g., sensitivity or subgroup analyses, meta-regression)	☒	☐	164-173
	15d	If quantitative synthesis is not appropriate, describe the type of summary planned	☐	☐	
**Meta-bias(es)**	16	Specify any planned assessment of meta-bias(es) (e.g., publication bias across studies, selective reporting within studies)	☐	☒	
**Confidence in cumulative evidence**	17	Describe how the strength of the body of evidence will be assessed (e.g., GRADE)	☐	☒	

For peer review only

BioMed Central The Open Access Publisher

For peer review only - http://bmjopen.bmj.com/site/about/guidelines.xhtml
